# Healthy lifestyles, screening, and breast cancer mortality in women with different risk of disease

**DOI:** 10.1093/oncolo/oyaf346

**Published:** 2025-10-10

**Authors:** Haomin Yang, Hongli Huang, Tao Zhang, Yanyu Zhang, Shuqing Zou, Pingxiu Zhu, Wei He, Yixuan Lin

**Affiliations:** Department of Epidemiology and Health Statistics, School of Public Health, Fujian Medical University, Fuzhou, Fujian 350122, China; Department of Medical Epidemiology and Biostatistics, Karolinska Institutet, Stockholm 17177, Sweden; Fuqing Center for Disease Control and Prevention, Fuzhou, Fujian 350399, China; Department of Clinical Medicine, Fujian Medical University, Fuzhou, Fujian 350122, China; Peking University Clinical Research Institute, Peking University Health Science Center, Beijing 100191, China; Department of Quality Control, Fujian Cancer Hospital, Fuzhou 350014, China; Peking University Clinical Research Institute, Peking University Health Science Center, Beijing 100191, China; Department of Medical Epidemiology and Biostatistics, Karolinska Institutet, Stockholm 17177, Sweden; The Children’s Hospital, and National Clinical Research Center for Child Health, Zhejiang University, Hangzhou, Zhejiang 310003, China; Department of Nutrition and Food Hygiene, School of Public Health, Zhejiang University, Hangzhou, Zhejiang 310058, China; Department of Epidemiology, University of Groningen, University Medical Center Groningen, Groningen 9713, The Netherlands

**Keywords:** breast cancer, screening, healthy lifestyles, mortality, risk predictors

## Abstract

**Background:**

Healthy lifestyles and screening are the two major interventions to prevent breast cancer mortality. However, their effects have not been compared simultaneously, and it remains unclear whether their benefits differ by women’ baseline breast cancer risk.

**Methods:**

A prospective cohort study was conducted using the UK biobank linked to national cancer registries, including 261 398 women aged 40-70. Hazard ratios (HRs) and population attributable fractions (PAF) for breast cancer mortality were estimated in relation to healthy lifestyle index (HLI) and screening status, using a Cox regression model. We further examined the interaction between HLI, screening and breast cancer risk predictors (Tyrer–Cuzick score and polygenic risk score [PRS], using highest and lowest 20% as high- and low-risk groups) for breast cancer mortality by likelihood ratio (LR) test.

**Results:**

Women with a high Tyrer–Cuzick score and PRS were associated with increased breast cancer mortality. HLI was inversely associated with breast cancer mortality (HR = 0.55, 95%CI = 0.42-0.72), and the effect did not differ statistically according to the risk scores. Women who participated in screening programs were at reduced risk of breast cancer mortality (HR = 0.70, 95% CI = 0.52-0.95), particularly among those women with high Tyrer–Cuzick score (*P* for interaction = .048). The fraction of breast cancer mortality cases that might be prevented (PAF) by screening was 14.27% (95%CI = 4.44-24.09) and by healthy lifestyle was 9.63% (95%CI = 3.10-16.16).

**Conclusions:**

Although women with a high PRS or Tyrer–Cuzick score is associated with increased breast cancer mortality, deaths are preventable through changing lifestyles and screening. These findings support personalized, risk-based strategies.

Implications for practice Healthy lifestyles and screening are two key interventions for preventing breast cancer mortality. However, the relative effectiveness of these two strategies has not been previously compared simultaneously. Our study shows that reductions in breast cancer mortality are more strongly attributed to screening than to modifiable lifestyle factors—with this effect being particularly pronounced among women with a high Tyrer–Cuzick score. These findings underscore the importance of personalized interventions for breast cancer prevention, placing particular emphasis on risk-based screening.

## Introduction

Breast cancer is the most common cancer among women in the world, causing approximately 670 000 deaths globally in 2022.^1^ The known risk factors for breast cancer include genetic mutations, family history, reproductive factors, and modifiable lifestyle factors,^2^ which contributes 3.8% to 20% of the risk.

To reduce the disease burden of breast cancer, several primary and secondary preventive interventions have been implemented. However, their effectiveness has not been compared simultaneously.^3-5^ The primary preventive interventions, which aim to eliminate risk factors for breast cancer, and enhance the immune system in the population,^6^ including a healthy diet, managing body weight, limiting alcohol consumption and engaging in physical activity. Previous studies suggested that a healthy lifestyle index (HLI)—a composite measure incorporating physical activity, body fat, smoking, dietary patterns, and alcohol consumption was associated with a lower risk of breast cancer.^7^^,^^8^ However, few studies have examined the impact of healthy lifestyles index on breast cancer mortality, and none of them considered women’s different genetic or hormonal predispositions to breast cancer.^9-11^

As the major secondary preventive intervention for breast cancer, the implementation of mammographic screening program helped to detected breast cancer at an earlier stage, and reduced the mortality ranging from 4% to 31% across European countries.^12^ The wide range in the reduced mortality is probably due to the organization and coverage of the screening program, and the baseline risk of breast cancer in different study populations.

To support more personalized screening strategies, breast cancer risk prediction models could be used to stratify individuals by risk, allowing the design of tailored screening strategies suitable for each group,^13^ such as the Gail model, the Tyrer–Cuzick model, and polygenic risk score (PRS) based on genome-wide significant single-nucleotide polymorphisms.^14^^,^^15^ However, it remains unclear whether risk-based screening could be a valuable strategy to maximize the benefit while minimize the harm.^16-19^

In this study, we will investigate how screening status and healthy lifestyle index are associated with the mortality of breast cancer among women with different predisposition to the disease risk. Specifically, whether adherence to screening program and a healthy lifestyle could influence the mortality of breast cancer among women with different baseline polygenic risk scores and Tyrer–Cuzick scores.

## Material and methods

### Study population

The UK biobank cohort (RRID: SCR_012815) was used to investigate the associations between screening status, healthy lifestyle, risk prediction scores and both the incidence and mortality of breast cancer. The UK biobank cohort included more than 500 000 participants (including 273 169 women) from 22 assessment centers across the United Kingdom between 2006 and 2010. Participants were aged from 40 to 70 years at enrolment. Aside from blood and urine sample, the participants also answered a touchscreen-based questionnaire on demographic, anthropometric, reproductive, lifestyle, and familial history. All the participants provided informed consent. The study was approved by the National Information Governance Board for Health and Social Care and the National Health Service North West Centre for Research Ethics Committee (Ref: 11/NW/0382, June 17, 2011).

### Screening status

In the touchscreen-based questionnaire, the participants were asked “Have you ever been for breast cancer screening (a mammogram)?” Response option included “Yes” “No” “Do not know” or “Prefer not to answer”. We defined those answered “Yes” as the screening group, while those who answered “No” as the non-screening group. Participants answered “Do not know” or “Prefer not to answer” were excluded from our studies.

### Genetic and hormonal risk prediction scores for breast cancer

Blood samples for almost all the UKB participants were genotyped using a custom-made Affymetrix chip, UK BiLEVE Axiom or the Affymetrix UKB Axiom array. Quality control and imputation for genotyped SNPs in the UK Biobank study have been described previously.^20^ In our study, 305 out of the 313 SNPs for breast cancer were used to calculate the weighted polygenic risk score of breast cancer, using the following formula:


$$\text{PRS = }\beta_{\mathrm{1x1}}+\beta_{\mathrm{2x2}}+\ldots .\beta_{kxk}+\beta_{nxn,}$$

where *β* is the per-allele log odds ratio (OR) of the associated risk allele for SNP, *xk* is the number of alleles for the same SNP (0, 1, 2), and *n* is the total number of the SNPs. The 313 SNPs is current the best performance PRS for breast cancer and have been used in the UK biobank for several studies.^21^^,^^22^

In the UK, the Tyrer–Cuzick model demonstrated better performance than the Gail model due to its inclusion of more comprehensive hormonal risk factors.^23^^,^^24^ The risk factors, including age at menarche, number of parity, age at first childbirth, age at menopause, previous atypical hyperplasia, previous lobular carcinoma in situ, height, and body mass index were collected for each woman either at the assessment center or with linkage to the National Health Service data or cancer register. These variables were entered into the model using the computer program developed for the IBIS-II breast cancer prevention study (IBIS, v7.0b).^25^

### Healthy lifestyle index

Healthy lifestyle index (HLI) was developed according to previous literature,^26^ incorporating factors such as diet, alcohol consumption, physical activity, body fat, and smoking. In UKB, a food frequency questionnaire was utilized to ask participants about the frequency and quantity of food intake over the previous 12 months. The frequency of alcohol consumption, physical activity, measurement of body fat (using body mass index [BMI] and Waist Circumference [WC]), and smoking are each assigned a score of 0 to 0.5 or 0 to 1, with the highest value (0.5 or 1) representing the highest category. The level of physical activity was evaluated by documenting the frequency and duration of walking, moderate-intensity, and vigorous-intensity exercises performed over the past week, using the International Physical Activity Questionnaire (IPAQ). The HLI was then constructed by summing up the scores for diet, alcohol consumption, physical activity, body fat, and smoking. Details about the construction of HLI the UK biobank was shown previously and in Supplementary Table S1.^7^

### Breast cancer mortality

The UK biobank cohort was linked to the national cancer registries in the United Kingdom to retrieve diagnoses of breast cancer with ICD-10 code C50. The main cause and date of death were retrieved from death certificates held by the National Health Service Registers. To study the association with breast cancer mortality, follow up of the cohort started from the date of participating in the UK biobank, and ended on the date of loss of follow-up, date of death or 31, December, 2021, whichever occurred first. Breast cancer patients diagnosed before their entrance into the UK biobank cohort were excluded from the study.

### Statistical analysis

The associations between HLI, screening status, risk predictors for breast cancer, and breast cancer mortality were examined using Cox regression model with age as the underlying time scale. For this analysis, as suggested by previous studies,^27^ women with the highest and lowest 20% of PRS and Tyrer–Cuzick score were considered as the high and low risk groups, while the medium 60% were those with the middle risk. HLI was also categorized similarly. In all the models, UKB assessment centers, educational qualifications (college or university degree/vocational qualification; national examination at ages 17-18 years; national examination at age 16 years; other qualifications were treated as missing) and ethnicity (White, black, Asia, mixed, and unknown) were adjusted, and we further adjusted for frequency of alcohol intake (≥once/day or <once/day), body mass index (BMI, categorized as <18.5, <25, <30, or ≥30 kg/m^2^), number of births (categorized as 0, 1, 2, or ≥3), age at menarche (categorized as <13, 13-15, >15, and <30 years), menopausal status (categorized as no, yes, not sure), age at first birth (categorized as <23, 23-27, >27 years, nulliparous/missing), ever use of oral contraceptive pill use (categorized as no or yes), and ever use of hormone replacement therapy (categorized as no or yes) when studying screening status. Missingness in the covariates were categorized as a separate category.

To examine the potential effect of risk-based screening, stratified analyses for breast cancer mortality were conducted according to screening status, PRS and Tyrer–Cuzick score. We further tested the interaction between preventive interventions (HLI and screening status) and risk predictors of breast cancer (PRS and Tyrer–Cuzick score), with an interaction term of these variables entered into the model and tested using the likelihood ratio (LR) test. In addition, the population attributable fractions of screening and health lifestyles for women’s mortality of breast cancer were estimated by different lifestyle factor and by baseline risk of breast cancer, using the AF package in R,^28^ adjusting for ethnicity, UKB centres, and education.

Considering that the organized screening program started from 50-year old in the UK and the risk predicting scores are mainly developed for population with European ancestry, we conducted sensitivity analyses including only those women > 50-year old, and including only those white women.

All analyses were performed using Stata MP version 17.0 (StataCorp LP, RRID: SCR_012763). All *P* values were two-sided, and a *P* value of less than .05 was considered statistically significant.

## Results

In our study, a total of 261 398 women participated in the cohort. The mortality rate of breast cancer was 0.20 per 1000 person-years. The participation rate of screening was high among the >60 years age group (99%) and lower among the 40-50 age group (26.5%). Women with a high participation rate of screening were those with family history (86.99%), previous smokers (83.67%), accept hormone replacement therapy (95.38%), and be postmenopausal (95.37%; Table 1).

**Table 1.** Baseline characteristics of the female participants of UKB by screening status.

Characteristics	Participate in screening (*N* = 206 456) No. (%)		Not participate (*N* = 54 942) No. (%)	
**Age, mean (SD), years**	59.43	(6.44)	46.54	(4.27)
**≤50-year old, *n* (%)**	16 758	(26.51)	46 447	(73.49)
**50- to 60-year old, *n* (%)**	82 864	(91.72)	7482	(8.28)
**>60-year old, *n* (%)**	106 834	(99.06)	1013	(0.94)
**Ethnicity, *n* (%)**				
**White**	196 997	(79.92)	49 505	(20.08)
**Mixed**	2758	(64.38)	1526	(35.62)
**Asian**	3409	(63.59)	1952	(36.41)
**Black**	2724	(60.25)	1797	(39.75)
**Place, *n* (%)**				
**England**	183 289	(79.18)	48 182	(20.82)
**Wales**	8458	(78.53)	2312	(21.47)
**Scotland**	14 709	(76.78)	4448	(23.22)
**Education, *n* (%)**				
**Lower qualification**	69 600	(74.97)	23 243	(25.03)
**Middle qualification**	35 820	(78.59)	9761	(21.41)
**Higher qualification**	97 327	(82.25)	21 000	(17.75)
**Townsend deprivation index**				
**By quartile, *n* (%)**				
**Lowest quartile**	53 229	(81.78)	11 857	(18.22)
**Second quartile**	53 377	(81.12)	12 427	(18.88)
**Third quartile**	51 989	(78.49)	14 249	(21.51)
**Highest quartile**	47 638	(74.48)	16 322	(25.52)
**PRS for BC**				
**Low**	41 209	(79.73)	10 479	(20.27)
**Middle**	120 629	(79.17)	31 736	(20.83)
**High**	38 269	(77.95)	10 826	(22.05)
**Tyrer**–**Cuzick score**				
**Low**	27 911	(52.74)	25 013	(47.26)
**Middle**	130 901	(83.21)	26 407	(16.79)
**High**	47 644	(93.12)	3522	(6.88)
**Family history of BC, *n* (%)**				
**No**	178 123	(78.07)	50 031	(21.93)
**Yes**	24 284	(86.99)	3633	(13.01)
**BMI, *n* (%), kg/m^2^**				
**<18.5**	1503	(75.26)	494	(24.74)
**18.5-24.9**	77 292	(76.26)	24 060	(23.74)
**25.0-29.9**	77 425	(81.22)	17 900	(18.78)
**≥ 30.0**	49 313	(80.17)	12 200	(19.83)
**Physical activity, by quartile, *n* (%)**				
**Lowest quartile**	39 252	(77.17)	11 610	(22.83)
**Second quartile**	40 297	(77.27)	11 856	(22.73)
**Third quartile**	39 777	(77.45)	11 581	(22.55)
**Highest quartile**	38 028	(79.74)	9662	(20.26)
**Smoking, *n* (%)**				
**Never**	120 661	(77.53)	34 966	(22.47)
**Previous**	68 072	(83.67)	13 285	(16.33)
**Current**	16 917	(72.10)	6545	(27.90)
**Alcohol consumption, *n* (%)**				
**Daily**	35 148	(83.82)	6787	(16.18)
**Drink more than once a week**	93 950	(77.73)	26 916	(22.27)
**Drink less than once a week or not at all**	77 209	(78.47)	21 178	(21.53)
**Menopause, *n* (%)**				
**No**	22 816	(36.03)	40 504	(63.97)
**Yes**	149 375	(95.37)	7250	(4.63)
**Hormone replacement therapy, *n* (%)**				
**No**	110 967	(68.92)	50 030	(31.08)
**Yes**	94 871	(95.38)	4599	(4.62)
**Contraception, *n* (%)**				
**No**	42 288	(86.41)	6651	(13.59)
**Yes**	163 648	(77.31)	48 035	(22.69)
**Menarche age, *n* (%)**				
**<12**	78 967	(80.09)	19 625	(19.91)
**13-15**	109 806	(78.51)	30 065	(21.49)
**16-29**	11 660	(77.60)	3365	(22.4)
**Children, *n* (%)**				
**0**	34 090	(69.74)	14 789	(30.26)
**1**	26 292	(75.33)	8611	(24.67)
**2-3**	93 431	(81.85)	20 713	(18.15)
**>3**	52 641	(82.94)	10 829	(17.06)
**Age of first birth, *n* (%)**				
**<23**	44 371	(86.14)	7140	(13.86)
**23-27**	60 661	(84.68)	10 977	(15.32)
**>27**	40 865	(75.35)	13 365	(24.65)
**Health lifestyle index**				
**Lower**	35 752	(77.77)	10 219	(22.23)
**Middle**	85 718	(77.50)	24 881	(22.5)
**High**	34 518	(78.96)	9197	(21.04)

Abbreviations: BC, breast cancer; PRS, polygenic risk score; SD, standard deviation; BMI, body mass index.

### Breast cancer mortality according to HLI, screening status and risk predictors

Women with a high PRS showed a 2.44 times higher mortality rates (95% CI: 1.90-3.14), compared to women with low PRS (Table 2). Similarly, those with a high Tyrer–Cuzick score exhibited an increased breast cancer mortality (HR = 1.62, 95% CI = 1.25-2.11), although the effect size was smaller than PRS. Interestingly, a 30% decrease in breast cancer mortality was observed among women who attended screening programs (HR = 0.70, 95% CI: 0.52-0.95), and adopting a healthy lifestyle was associated with a lower breast cancer mortality (HR = 0.55, 95% CI: 0.42-0.72).

**Table 2.** HRs of different factor on breast cancer mortality.

	No. of participants	No. of cases	HR (95% CI)^a^	*P* value
**PRS categories**				
**Low**	51 813	89	1 [reference]	
**Middle**	152 851	361	1.40 (1.11-1.76)	.005
**High**	49 334	198	2.44 (1.90-3.14)	<.001
***P* for trend**				<.001
**Tyrer**–**Cuzick score categories**				
**Low**	53 321	94	1 [reference]	
**Middle**	157 910	370	1.02 (0.81-1.30)	.851
**High**	51 224	199	1.62 (1.25-2.11)	<.001
***P* for trend**				<.001
**Screening status**				
**No**	54 942	109	1 [reference]	
**Yes**	206 456	549	0.61(0.46-0.80)	.001
**Health lifestyle index**				
**Low**	46 052	149	1 [reference]	
**Middle**	110 801	278	0.75 (0.62-0.92)	.005
**High**	43 806	80	0.53 (0.41-0.70)	<.001
***P* for trend**				<.001

Abbreviations: BC, breast cancer; CI, confidence interval; HR, hazard ratio; PRS, Polygenic risk score.

a Adjusted for ethnicity, UKB centres and education qualifications.

**Figure 1.**
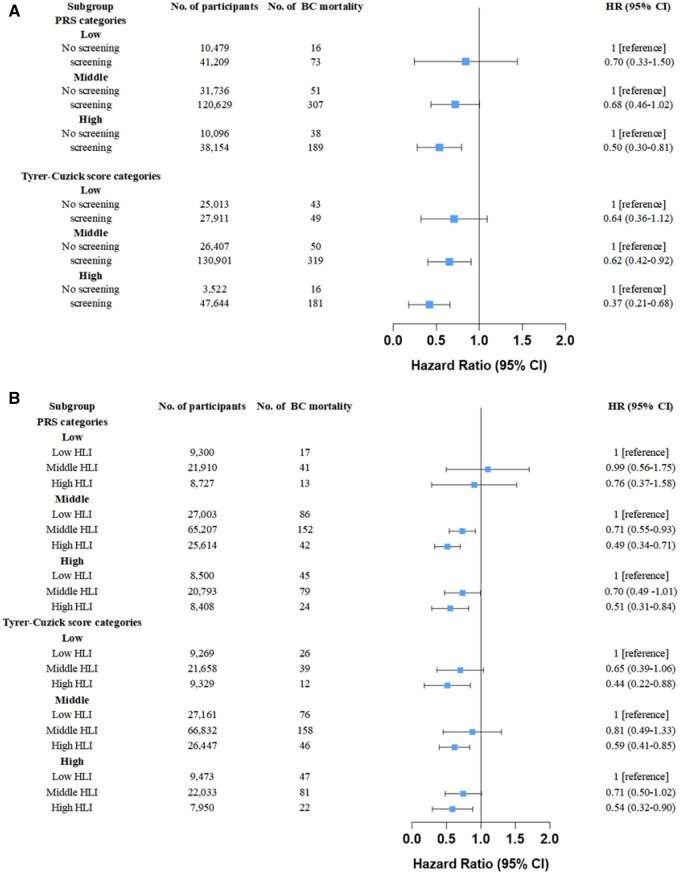
The effect of screening and healthy lifestyles on breast cancer mortality in different risk status. (A) The effect of screening. (B) The effect of healthy lifestyles. The models were adjusted for ethnicity, UKB centers and education qualifications.

When further investigating the effect of screening status on breast cancer mortality across different baseline risk levels (Figure 1A), the protective effect increased as PRS categories shift from low (HR = 0.70, 95%CI = 0.33-1.50) to high (HR = 0.50, 95%CI = 0.30-0.81), and screening was significantly associated with a lower risk of mortality in women in the high PRS group, while no significant association was observed in the middle or low risk subgroups. A similar trend was observed for Tyrer–Cuzick score, with significant associations observed among the middle (HR = 0.62, 95% CI = 0.42-0.92) and high (HR = 0.37, 95% CI = 0.21-0.68) and risk groups. Further interaction tests identified a statistically significant interaction between Tyrer–Cuzick score and screening status (*P* = .048), although no interaction was identified for PRS.

The effect of healthy lifestyles on breast cancer mortality was similar among women with different baseline risk of breast cancer identified by PRS or Tyrer–Cuzick score, despite that the association was not statistically significant among women with low PRS (HR = 0.76, 95% CI = 0.37-1.58; Figure 1B).

In the sensitivity analysis among women older than 50, the associations between risk predictors, lifestyle factors and breast cancer risk and mortality remained statistically significant, except that the effect of screening on breast cancer mortality was attenuated to 0.74 and was no longer significant (Supplementary Table S2). Estimates in the white women did not change substantially. (Supplementary Table S3).

### Population attributable fractions

**Figure 2.**
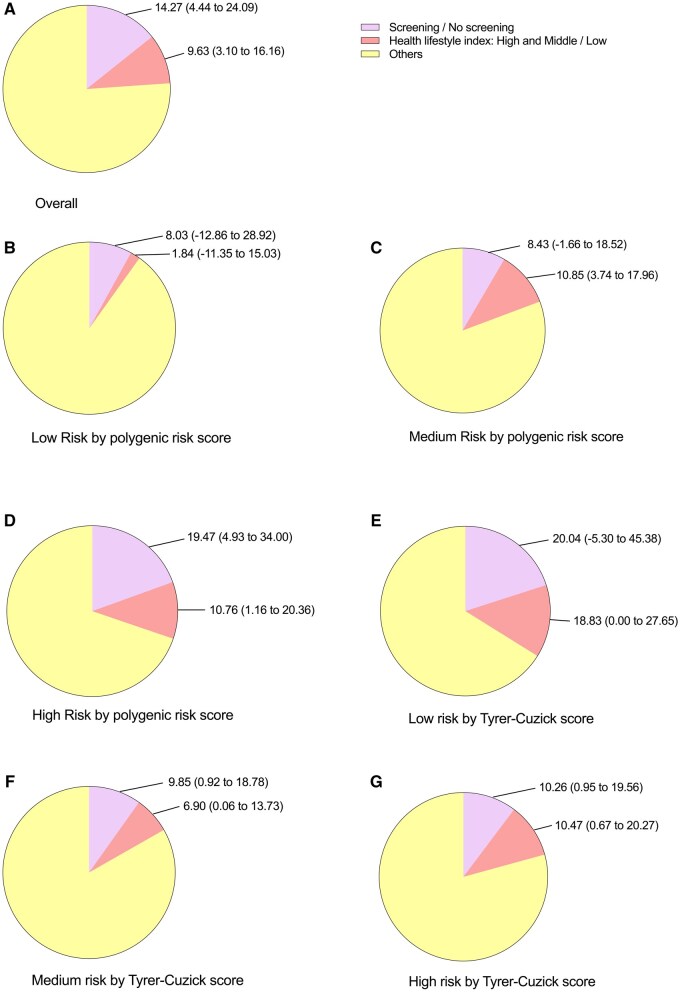
Population-attributable fraction (PAF) by screening and health lifestyles for breast cancer mortality among women with different risk for breast cancer. The models were adjusted for ethnicity, UKB centers and education qualifications.

During the follow-up period, 14.27% (95% CI: 4.44-24.09) of the breast cancer mortality cases were attributed to lack of screening, while 9.63% (95% CI: 3.10-16.16) was attributed to unhealthy lifestyle factor, especially for smoking with a 5.24% attributable fraction (Supplementary Table S4). When stratified by risk predictors, 19.47% (95% CI: 4.93-34.00) of breast cancer mortality case could be attributed to screening in women with high PRS, which is the largest significant attributed fraction observed among the different risk groups (Figure 2).

## Discussion

In this population-based cohort study, we identified significant associations between PRS and Tyrer–Cuzick score with breast cancer mortality. Participation in screening was associated with a lower risk of breast cancer death, particularly among women with a high Tyrer–Cuzick score. Adherence to a healthy lifestyle may also reduce breast cancer mortality, while the effect did not differ according to the risk profile. In addition, the reduction in breast cancer mortality attributed to screening was greater than that attributed to changes in lifestyle factors.

In this study we found that PRS and Tyrer–Cuzick score are reliable predictors of breast cancer mortality. Similar to our findings, several large population-based studies demonstrated that PRS is a robust indicator of breast cancer risk.^29^^,^^30^ A recent study further suggested a combination of Tyrer–Cuzick score and PRS may improve the accuracy of breast cancer prediction,^24^ supporting their integration into the risk assessment of women.

Although women cannot alter their genetic and reproductive factor for breast cancer, our study demonstrated that a healthy lifestyle may reduce breast cancer mortality. Moreover, this reduction of breast cancer mortality was observed among women with different risk profiles of breast cancer, suggesting it as a universally beneficial intervention. Among the different lifestyle factors, smoking accounted for the largest portion of deaths attributable to lifestyle. This large proportion was probably due to both the increased risk and decreased survival rates for breast cancer among female smokers.^31^ Consequently, policymakers may therefore balance the benefit of tobacco tax revenue with the imperative to safeguard public health. Consistent with our findings, similar concern applies to BMI, as a systematic review reported that each 5 kg/m^2^ increase was associated with a 2% higher breast cancer risk,^32^ and chronic obesity was associated with increased cancer mortality.^33^ Furthermore, evidence from UK Biobank^34^ and a systematic review^35^ indicates that higher physical activity is associated with reduced breast cancer risk and lower mortality among survivors.

Attending to screening programs is another approach to reduce breast cancer mortality. Findings in our study are consistent with previous study showing that screening can reduce around 20% of mortality among women at the average risk.^36^ The attributable fraction of screening for breast cancer mortality was even larger than any lifestyle factors, suggesting it to be a better intervention for mortality reduction. Nonetheless, ongoing debates exist as to whether mortality reduction is due to screening itself or to advances in treatment.^37^ Additionally, screening may cause overdiagnosis—around 80% of ductal carcinoma in situ (DCIS) may never progress to invasive breast cancer.^38^ Although studies already indicated that in DCIS was still associated with an increased long-term risk of breast cancer mortality,^39^ distinguishing DCIS prone to invasive disease together with the screening program might be important for further studies.^40^

When evaluating PRS and Tyrer–Cuzick score under a screening context, we found that women with high Tyrer–Cuzick scores who participated in screening experience a reduced mortality by 60% compared to those who did not attend. Moreover, an interaction was identified between screening and Tyrer–Cuzick score. This finding suggested that Tyrer–Cuzick scores may be a valuable indicator for risk-based screening, with women at higher risk benefit more from screening programs.

In the sensitivity analysis, the associations between risk predictors, lifestyle factors and mortality were almost retained among white ethnicity women. This was because white ethnicity took 94% of the entire population. In many studies, researchers pointed out that genetic risks are not consistence in different ethnicities,^41-43^ and PRS for breast cancer developed among European population is not applicable to African populations, limiting the generalizability of our results.^44^

The primary strength of our study is the inclusion of comprehensive factors to assess the effectiveness of each predictor and compare the effect of primary and secondary preventive measurements for breast cancer mortality. However, our study also had some limitations. First, information on tumour node metastasis (TNM) and hormone receptor status classification was unavailable, preventing us from analysing the influence of tumor characteristics on breast cancer mortality. Second, data such as screening behavior and lifestyle factors were self-reported at baseline, and may have changed during the follow-up time. HLI is a simplified tool for measuring lifestyle behavior, but it may miss information on the individual situation, while screening status could not capture information on screening interval and frequency. All these may potentially introduce misclassification and underestimate the true effects of screening and HLI. Third, UK Biobank may have “healthy volunteer” bias, as participants tend to be more affluent, healthier and less ethnically diverse than the general population, underestimating the absolute risk effect on mortality. However, biased cumulative cancer risks do not necessarily bias the association between exposure and cancer and relative risk estimates remain reliable in large-scale studies like the UK Biobank.^45^

## Conclusions

Although women with a high PRS or Tyrer–Cuzick score are associated with increased breast cancer mortality, this can be reduced through changing lifestyles and screening. Attending screenings is a significant modifiable factor that can reduce breast cancer mortality, with a larger attributable fraction than changing lifestyles, and the effect is stronger among women with a high Tyrer–Cuzick score. These findings support the development of personalized interventions for breast cancer prevention and risk-based screening programs.

## Supplementary Material

oyaf346_Supplementary_Data

## Data Availability

Data from UK Biobank (http://www.ukbiobank.ac.uk/) are available to researchers upon application.This research was conducted using the UK Biobank Resource under Application 61083.
