# Supplementary material for: Healthy lifestyles, screening, and breast cancer mortality in women with different risk of disease
Source: Oncologist. 2025 Oct 10;30(11):oyaf346. doi: 10.1093/oncolo/oyaf346 (PMC12605817; doi:10.1093/oncolo/oyaf346)
Supplement: oyaf346_Supplementary_Data [file oyaf346_supplementary_data.zip › Supplementary_Data.docx]

**Supplemental materials**

**Supplementary Table 1**. The construction of Health Lifestyle Index

**Supplementary Table 2**. HRs of different factors on breast cancer mortality in women older than 50 years

**Supplementary Table 3.** HRs of different factors on breast cancer mortality in white women

Supplementary Table 4. Population-attributable fraction (PAF) by screening and health lifestyles for breast cancer mortality among women with different risk for breast cancer

**Supplementary Table 1**. The construction of Health Lifestyle Index

| WCRF/AICR recommendations | Items | Categories | Score |
| --- | --- | --- | --- |
| 1. the diet rich in whole grains, vegetables, fruits, legumes* | Total intake of fruit and vegetable | <3 servings/day | 0 |
|  |  | ≥3~5 servings /day | 0.25 |
|  |  | ≥5 servings /day | 0.5 |
|  |  | （1 serving：1 piece of fresh fruit; 5 pieces of dried fruits; Boiled/Lettuce 3 tablespoons） |  |
|  | Whole grains intake | ≤ 2 servings /day | 0 |
|  |  | >2~<5.5 servings /day | 0.25 |
|  |  | ≥5.5 servings /day | 0.5 |
|  |  | （1 serving：bran/oatmeal/cereal congee 1 bowl/day; whole wheat bread 1 slice/day） |  |
| 1. red/processed meats in moderation | Red meat intake | >4 times/week | 0 |
|  |  | 2~4 times/week | 0.25 |
|  |  | ≤1 time/week | 0.5 |
|  | Red meat intake | >4 times/week | 0 |
|  |  | 2~4 times/week | 0.25 |
|  |  | ≤1 time/week | 0.5 |
| 3）Drink in moderation | Frequency of drinking | never | 1 |
|  |  | <5 times/week | 0.5 |
|  |  | ≥5 times/week | 0 |
| 4）More exercise | Physical activity | <600 MET-minute/week | 0 |
|  |  | ≥600~<3000 MET- minute/week | 0.5 |
|  |  | ≥3000 MET- minute/week | 1 |
| 5）Healthy weight | BMI ** | 18.5-24.9 kg/m2 | 0.5 |
|  |  | 25.0-29.9 kg/m2 | 0.25 |
|  |  | >29.9 kg/m2 | 0 |
|  | Waist circumference** | <80 cm | 0.5 |
|  |  | >80~<88 cm | 0.25 |
|  |  | ≥88 cm | 0 |
| 6）Limit consumption of fast foods and other processed foods high in fat, starches, or sugars | These five recommendations were not included in the construction of HLI * | | |
| 7）Limit consumption of sugary drinks |  |  |  |
| 8）No supplements used for cancer prevention |  |  |  |
| 9）Breastfeeding (for mothers) |  |  |  |
| 10）After cancer diagnosis, follow the recommended treatment guidelines |  |  |  |
| Smoking isn’t included in WCRF/AICR | smoking | Never | 1 |
|  |  | Former | 0.5 |
|  |  | Current | 0 |

* UK Biobank food frequency table does not include information on legume intake and the five recommended items mentioned above.

** BMI and waist circumference scores are reversed among premenopausal women (women under 55 years old or self-reported as premenopausal are classified as premenopausal women, while women over 55 years old or self-reported as postmenopausal are classified as postmenopausal women).

The composition and scoring of each component of HLI refer to Arthur et al. Adherence to Healthy Lifestyle Behavior, and Risk of Invasive Breast Cancer Among Women in the UK Biobank. *J Natl Cancer Inst*. 2020;112(9):893-901.

| **Supplementary Table 2. HRs of different factors on breast cancer mortality in women older than 50 years** | | | | |
| --- | --- | --- | --- | --- |
|  | **No. of  participants** | **No. of  cases** | **HR (95% CI)^a^** | **P value** |
| **PRS categories** |  |  |  |  |
| Low | 39,994 | 70 | 1 [reference] |  |
| Middle | 115,999 | 306 | 1.52 (1.17-1.97) | 0.002 |
| High | 36,514 | 157 | 2.54 (1.91-3.37) | < 0.001 |
| P for trend |  |  |  | < 0.001 |
|  |  |  |  |  |
| **Tyrer-Cuzick score categories** |  |  |  |  |
| Low | 23,586 | 46 | 1 [reference] |  |
| Middle | 129,608 | 319 | 1.17 (0.86-1.60) | 0.328 |
| High | 45,527 | 179 | 1.80 (1.30-2.51) | < 0.001 |
| P for trend |  |  |  | < 0.001 |
|  |  |  |  |  |
| **Screening status** |  |  |  |  |
| No | 8,495 | 18 | 1 [reference] |  |
| Yes | 189,698 | 523 | 0.74 (0.45-1.19) | 0.214 |
|  |  |  |  |  |
| **Health lifestyle index** |  |  |  |  |
| Low tertile | 34,186 | 125 | 1 [reference] |  |
| Middle tertile | 81,767 | 222 | 0.71 (0.57-0.89) | 0.003 |
| High tertile | 33,082 | 59 | 0.46 (0.34-0.62) | < 0.001 |
| P for trend |  |  |  | < 0.001 |
| Abbreviations: BC, breast cancer; CI, confidence interval; HR, hazard ratio; PRS, Polygenic risk score. ^a^Adjusted for ethnicity, UKB centers, and education qualifications. | | | | |

| **Supplementary Table 3. HRs of different factors on breast cancer mortality in white women** | | | | |
| --- | --- | --- | --- | --- |
|  | **No. of  participants** | **No. of  cases** | **HR (95% CI)^a^** | **P value** |
| **PRS categories** |  |  |  |  |
| Low | 50,593 | 88 | 1 [reference] |  |
| Middle | 145,371 | 347 | 1.38 (1.09-1.75) | 0.007 |
| High | 43,440 | 177 | 2.39 (1.85-3.08) | < 0.001 |
| P for trend |  |  |  | < 0.001 |
|  |  |  |  |  |
| **Tyrer-Cuzick score categories** |  |  |  |  |
| Low | 46,865 | 83 | 1 [reference] |  |
| Middle | 150,268 | 349 | 1.02 (0.79-1.31) | 0.893 |
| High | 49,758 | 194 | 1.64 (1.25-2.15) | < 0.001 |
| P for trend |  |  |  |  |
|  |  |  |  | < 0.001 |
| **Screening status** |  |  |  |  |
| No | 49,505 | 99 | 1 [reference] |  |
| Yes | 196,997 | 523 | 0.59 (0.44-0.80) | < 0.001 |
|  |  |  |  |  |
| **Health lifestyle index** |  |  |  |  |
| Low | 44,364 | 141 | 1 [reference] |  |
| Middle | 105,123 | 265 | 0.77 (0.63-0.95) | 0.012 |
| High | 40,292 | 76 | 0.56 (0.42-0.74) | < 0.001 |
| P for trend |  |  |  | < 0.001 |
| Abbreviations: BC, breast cancer; CI, confidence interval; HR, hazard ratio; PRS, Polygenic risk score. ^a^Adjusted for ethnicity, UKB centers, and education qualifications. | | | | |

| Supplementary Table 4. Population-attributable fraction (PAF) by screening and health lifestyles for breast cancer mortality among women with different risk for breast cancer | | | | | | | |
| --- | --- | --- | --- | --- | --- | --- | --- |
|  | | **PAF (%)** | | | | **95% CI** | |
| **Overall** | |  | | | |  | |
| **Screening / No screening** | | 14.27 | | | | 4.44 to 24.09 | |
| **Health lifestyle index**: High and Middle / Low | | 9.63 | | | | 3.10 to 16.16 | |
| **Diet score:**≥1 / <1 | | | 0.90 | | -3.90 to 5.69 | | |
| **Current Smoking status**: No / Yes | | | 5.24 | | 1.48 to 8.99 | | |
| **Alcohol intake:** No / Yes | | | 0.15 | | -2.39 to 2.68 | | |
| **BMI, kg/m^2^ :** <25 /≥ 25 | | | 4.12 | | -9.37 to 17.61 | | |
| **Physical activity, MET-minutes/week:** ≥600 / <600 | | | 2.49 | | -1.97 to 6.94 | | |
| **Risk by polygenic risk score** | |  | | | |  | |
| **Low risk by PRS** | |  | | | |  | |
| **Screening / No screening** | | 8.03 | | | | -12.86 to 28.92 | |
| **Health lifestyle index:** High and Middle / Low | | 1.84 | | | | -11.35 to 15.03 | |
| **Middle risk by PRS** | |  | | | |  | |
| **Screening / No screening** | | 8.43 | | | | -1.66 to 18.52 | |
| **Health lifestyle index:** High and Middle / Low | | 10.85 | | | | 3.74 to 17.96 | |
| **High risk by PRS** | |  | | | |  | |
| **Screening / No screening** | | 19.47 | | | | 4.93 to 34.00 | |
| **Health lifestyle index:** High and Middle / Low | | 10.76 | | | | 1.16 to 20.36 | |
| **Risk by Tyrer-Cuzick score** | |  | | | |  | |
| **Low risk by Tyrer-Cuzick score** |  | | |  | | | |
| **Screening / No screening** | 20.04 | | | | | | -5.30 to 45.38 |
| **Health lifestyle index:** High and Middle / Low | 13.83 | | | | | | 0.00 to 27.65 |
| **Middle risk by Tyrer-Cuzick score** |  | | | | | |  |
| **Screening / No screening** | 9.85 | | | | | | 0.92 to 18.78 |
| **Health lifestyle index:** High and Middle / Low | 6.9 | | | | | | 0.06 to 13.73 |
| **High risk by Tyrer-Cuzick score** |  | | | | | |  |
| **Screening / No screening** | 10.26 | | | | | | 0.95 to 19.56 |
| **Health lifestyle index:** High and Middle / Low | 10.47 | | | | | | 0.67 to 20.27 |
| Abbreviation: BC, breast cancer; CI, confidence interval. Adjusted for ethnic, UKB centers, and education.  Abbreviation: BC, breast cancer; CI, confidence interval.  Adjusted for ethnicity, UKB centres, and education qualifications. | | | | | | | |
